# Genome-wide analysis of the C3H zinc finger family reveals its functions in salt stress responses of *Pyrus betulaefolia*

**DOI:** 10.7717/peerj.9328

**Published:** 2020-06-11

**Authors:** Chunxiao Liu, Xiaoyang Xu, Jialiang Kan, Zong ming Cheng, Youhong Chang, Jing Lin, Hui Li

**Affiliations:** 1 Institute of Pomology, Jiangsu Key Laboratory for Horticultural Crop Genetic Improvement, Jiangsu Academy of Agricultural Sciences, Nanjing, Jiangsu, China; 2 Institute of Botany, Jiangsu Province and Chinese Academy of Sciences, Nanjing, Jiangsu, China; 3 Department of Plant Sciences, University of Tennessee—Knoxville, Knoxville, TN, United States of America

**Keywords:** *Pyrus betulaefolia*, C3H gene family, Genome-wide, Evolution, Salt stress response

## Abstract

Transcription factors regulate gene expression in response to various external and internal cues by activating or suppressing downstream genes. Significant progress has been made in identifying and characterizing the Cysteine3Histidine (C3H) gene family in several dicots and monocots. They are characterized by their signature motif of three cysteine and one histidine residues, and reportedly play important roles in regulation of plant growth, developmental processes and environmental responses. In this study, we performed genome-wide and deep analysis of putative C3H genes, and a total of 117 PbeC3H members, were identified in *P. betulaefolia* and classified into 12 groups. Results were supported by the gene structural characteristics and phylogenetic analysis. These genes were unevenly distributed on 17 chromosomes. The gene structures of the C3H genes were relatively complex but conserved in each group. The C3H genes experienced a WGD event that occurred in the ancestor genome of *P. betulaefolia* and apple before their divergence based on the synonymous substitutions (*K*s) values. There were 35 and 37 pairs of paralogous genes in the *P. betulaefolia* and apple genome, respectively, and 87 pairs of orthologous genes between *P. betulaefolia* and apple were identified. Except for one orthologous pairs PbeC3H66 and MD05G1311700 which had undergone positive selection, the other C3H genes had undergone purifying selection. Expression profiles showed that high salinity stress could influence the expression level of C3H genes in *P. betulaefolia*. Four members were responsive to salt stress in roots, nine were responsive to salt stress in leaves and eight showed inhibited expression in leaves. Results suggested important roles of *PbeC3H* genes in response to salt stress and will be useful for better understanding the complex functions of the C3H genes, and will provide excellent candidates for salt-tolerance improvement.

## Introduction

The zinc finger protein (ZFP) is one of the largest and specific transcription factor families in plants. Members are characterized by common zinc finger (Znf) motifs in which cysteines and/or histidines coordinate with a few zinc ions to form the local peptide structures (Laity, Lee & Wright, 2001; Hall, 2005). The ZFPs are classified into at least 14 gene families based on their structural and functional characteristics, among which RING finger, DOF, LIM, AP2/EREBP and WRKY have been identified to play important roles in plant growth and response to biotic and abiotic stresses (Arnaud et al., 2007; Huang et al., 2015; Ma et al., 2015; Liu & Zhang, 2017). The ZFPs have been categorized into 10 groups (C2H2, C2HC, C2HC5, C2C2, C3H, C3HC4, C4, C4HC3, C6 and C8) based on the number of cysteine and histidine residues and the number of amino acids in the spacer region (Moore & Ullman, 2003). The ZFPs are recognized as master regulators of several downstream genes involved in multiple biological processes, such as development, morphogenesis, signal transduction and environmental stress responses (Takatsuji, 1998; Stege et al., 2002; Yang et al., 2014).

Among ZFPs, Cysteine3Histidine (C3H) ZFPs are characterized by a Znf motif consisting of three cysteines and one histidine coordinated by a zinc cation (Bogamuwa & Jang, 2014), which are conserved mRNA-binding proteins in many eukaryotes (Blackshear, 2002; Wang et al., 2008). Members of this family have been found in eukaryotes ranging from yeast to human (Thompson et al., 1996; De et al., 1999; Carrick, Lai & Blackshear, 2004). A typical C3H protein contains 1-6 C3H-type Znf motifs. Based on the different numbers of amino acid spacers between cysteines and histidines in the C3H motif, a consensus sequence for these motifs was defined as C-X_4−17_-C-X_4−6_-C-X_3_-H (X represents any amino acid) (Peng et al., 2012; Yuan et al., 2015). A whole-genome screen for C3H proteins in *Arabidopsis*, rice and maize found two common C3H motifs (C-X_7_-C-X_5_-C-X_3_-H and C-X_8_-CX_5_-C-X_3_-H) (Peng et al., 2012).

Increasing evidence has revealed that C3H proteins participate in regulation of growth, developmental processes and environmental responses in plants. In *Arabidopsis*, HUA1, the first reported C3H-type ZFP with six tandem C3H motifs, was identified as an RNA-binding protein and likely participates in a new regulatory mechanism for flower development (Li, Jia & Chen, 2001). The C3H ZFP *AtPEI1* plays an important role during *Arabidopsis* embryogenesis (Li & Thomas, 1998) and interaction of *FES1* with *FRI* and *FRL1* genes, which is required to promote the winter-annual habit in *Arabidopsis* (Schmitz et al., 2005). A rice C3H ZFP gene, *OsLIC*, mediates rice architecture via brassinosteroid signaling (Wang et al., 2008). Another rice C3H ZFP gene, *OsDOS*, is involved in delaying leaf senescence (Kong et al., 2006). Additionally, studies have shown that some C3H ZFPs are involved in abiotic stress, especially in salt stress responses. For example, transgenic *Arabidopsis* overexpressing *AtC3H17* were more tolerant under NaCl and methyl viologen treatment than the wild type (Seok et al., 2018). A cotton Znf gene, *GhZFP1*, enhanced tolerance to salt stress and resistance to fungal disease in transgenic tobacco (Guo et al., 2009). Arabidopsis genes *AtTZF10* (*AtSZF2/AtC3H29*) and *AtTZF11* (*AtSZF1/AtC3H47*) are involved in salt stress responses (Sun et al., 2007). Over expression of *AtTZF2* and *AtTZF3* caused enhanced tolerance to drought, oxidative and salt stress. In contrast, the antisense or RNAi plants exhibited reduced to salt stress (Huang et al., 2011; Huang et al., 2012; Lee et al., 2012). In cotton, compared with the WT, GhZFP1 over expression plants show increased salt tolerance, due to changes in Na^+^ and K^+^ homeostasis (Guo et al., 2009). Moreover, native *CCCH-ZFP* gene *AtTZF1* over expression in *Arabidopsis* (Lin et al., 2011; Zang et al., 2016), in rice (Sun et al., 2010; Jan et al., 2013) and in broccoli (Jiang et al., 2017) resulted in higher salt stress-tolerance response.

Abiotic stresses are key environmental threats that constrain plant growth, development and yield (Cramer et al., 2011). Among various abiotic stresses, salinity, drought and extreme temperatures are major factors (Luo et al., 2015). The C3H members play important roles in plant response to the environment. A number of researchers have elucidated the association of C3Hs with diverse plant growth regulatory processes via genome-wide analysis in *Arabidopsis* and rice (Wang et al., 2008), poplar (Chai et al., 2012), maize (Peng et al., 2012), switchgrass (Yuan et al., 2015), alfalfa (Zhang et al., 2013), citrus (Liu et al., 2014), tomato (Xu, 2014), grape vine (Wang, Zhong & Cheng, 2014), chickpea (Pradhan et al., 2017), banana (Mazumdar et al., 2017) and cabbage (Rameneni et al., 2018).

Pears (*Pyrus* spp. L.) are one of the most important fruit crops in the *Rosaceae* family. They are the third most important fruit crop in temperate zones, after grape and apple (Chang et al., 2012; Wu et al., 2013). Pear production is quite limited due to the spread of soil salinization and *Pyrus betulaefolia* Bunge, a Chinese native wild pear species, is commonly used as a rootstock in pear orchards to improve abiotic stress tolerance (Okubo & Sakuratani, 2000; Matsumoto et al., 2007; Chang et al., 2012; Dong et al., 2019). Although the characterizations of C3H Znf proteins have been reported in some other species of plants and animals, their functions are poorly understood in *P. betulaefolia*. In this study, we performed a comprehensive analysis of 117 members of the C3H Znf gene family in *P. betulaefolia*, including phylogenetic analysis, chromosomal location, gene structure, gene evolution and expression profiles in various organs and under salt treatment. Our investigation should provide an important foundation for future cloning and functional studies of PbeC3H proteins and excellent candidates for *P. betulaeolia* improvement.

## Materials and Methods

### Identification of C3H proteins

The latest version of the *P. betulaefolia* genome and protein sequences were downloaded from BioProject (PRJNA529328) of NCBI (https://www.ncbi.nlm.nih.gov/bioproject/) (Dong et al., 2019). The C3H genes of cultivated pear *P. bretschneideri* were got from genome (version v1.1) project (http://peargenome.njau.edu.cn/). The *Malus domestica* C3H gene files were downloaded from the Genome Database for *Rosaceae* (https://www.rosaceae.org/). The Hidden Markov Model (HMM) profiles of C3H domains (PF00642) were obtained from the Pfam database (http://pfam.xfam.org/), and it was employed as a query to identify all possible C3H proteins using HMMER (V3.0) software (Finn, Clements & Eddy, 2011). Then the motif was confirmed using inline tool SMART (http://smart.emblheidelberg.de/) and MOTIF Search (https://www.genome.jp/tools/motif/).

### Sequence analyses

Protein properties, including three fields (length, molecular weight and isoelectric point) of each PbeC3H protein were calculated by online ExPasy program (http://www.expasy.org/tools/). The motif analyses of the PbeC3Hs were detected using MEME online software (http://meme-suite.org/tools/meme/) with the default parameter settings, the width of motifs was set from 6 to 50, and the number of motifs was 2 to 15. The gene structures of the PbeC3Hs were parsed from the general feature format (GFF) files of the *P. betulaefolia* genome database, and diagrams of the exon-intron structures were drawn using the online program Gene Structure Display Server (GSDS, http://gsds.cbi.pku.edu.cn/).

### Mapping C3H genes on chromosomes

Positional information of all the C3Hs was parsed from the *P. betulaefolia* genome; the locations of them was drafted using MapInspect software (version 1.0) (http://mapinspect.software.informer.com/).

### Phylogenetic analysis of C3H proteins

All obtained proteins were aligned using ClustalX2.0 (Larkin et al., 2007). Phylogenetic trees were generated using the Maximum Likelihood method in MEGA7 (Kumar, Stecher & Tamura, 2016) software, and the reliability of the interior branches was assessed with 1,000 bootstrap re-samplings.

### *K*s calculation and divergence time estimation of homologous gene pairs

The ratio of non-synonymous substitutions (*K*a)/synonymous substitutions (*K*s) was evaluated to determine homologous relationships and divergence time of C3H genes. *K*a and *K*s values, and the ratio of *K*a/*K*s of C3H homologous gene pairs in *P. Betulaefolia* or in apple, and orthologous gene pairs between *P. betulaefolia* and apple were calculated using DnaSP v5 (Librado & Rozas, 2009). The approximate divergence time of the C3H homologous gene pairs in *P. betulaefolia*, apple, or between them were calculated based on the formula T = *K*s/2*λ* assuming a clock-like rate (*λ*) of 9.26 synonymous substitutions per 10^9^ years (Wu et al., 2013). A syntenic diagram was constructed using Circos software (Krzywinski et al., 2009).

### Plant material and treatments

The plants of *P. betulaefolia* were planted in soil from the tissue cultures of one-month seedlings. The seedlings were grown in a growth chamber with fixed chamber condition (light/dark cycle:14 h at 25 °C/10 h at 23 °C; 65% relative humidity). About 45 days later, at the eight-leaf stage, the roots of the *P. betulaefolia* plants were immersed into solution with 200 mM NaCl and the deionized water as controls as the treatment before (Li et al., 2017). Roots, stems and leaves were collected at 0 h (just prior to the application of the salt treatment) 12 h, 24 h, 48 h and 72 h after the salt treatment. Samples were immediately frozen in liquid nitrogen and stored at −80 °C. The experiments were repeated three times, and each experiment was comprised of 6 plants per treatment. The presented data represents the mean ± the standard error of three biological replicates.

### RNA isolation and quantitative reverse transcription PCR

Total RNA was isolated using a plant RNA purification kit (MoLFarming, Cat. No. RK16-50 T, Nanjing, China) from leaf and root tissues according to the manufacturer’s instructions. The expression of PbeC3H genes was analyzed using a BIO-RAD CFX Connect Real-Time system (BIO-RAD, California, USA) with the SYBR Green Master Mix (TSINGKE, Beijing, China). Gene-specific primers were designed based on the gene sequences using Primer Premier 5.0 (Carnegie Institute of Washington, Washington, USA). *EF1α* (*GWHPAAYT007384*) of *P. betulaefolia* was used as internal controls for normalization (Liu et al., 2018). The efficiency of the RT-qPCR primers was tested using both RT-qPCR and polyacrylamide gel electrophoresis. And the specific primers then selected for further analysis. The amplification parameters were as follows: 95 °C hold for 10 min, followed by 40 cycles at 95 °C for 15 s, 60 °C for 15 s, and 72 °C for 15 s. Nonspecific products were identified by inspecting melting curves. Experiments for three technical replicates for each biological replicate were carried out. A *t*-test was used for statistical analysis. The primers used in this article were list in Table S1.

## Results

### Identification and characterization of the *PbeC3H* family genes

**Figure 1:**
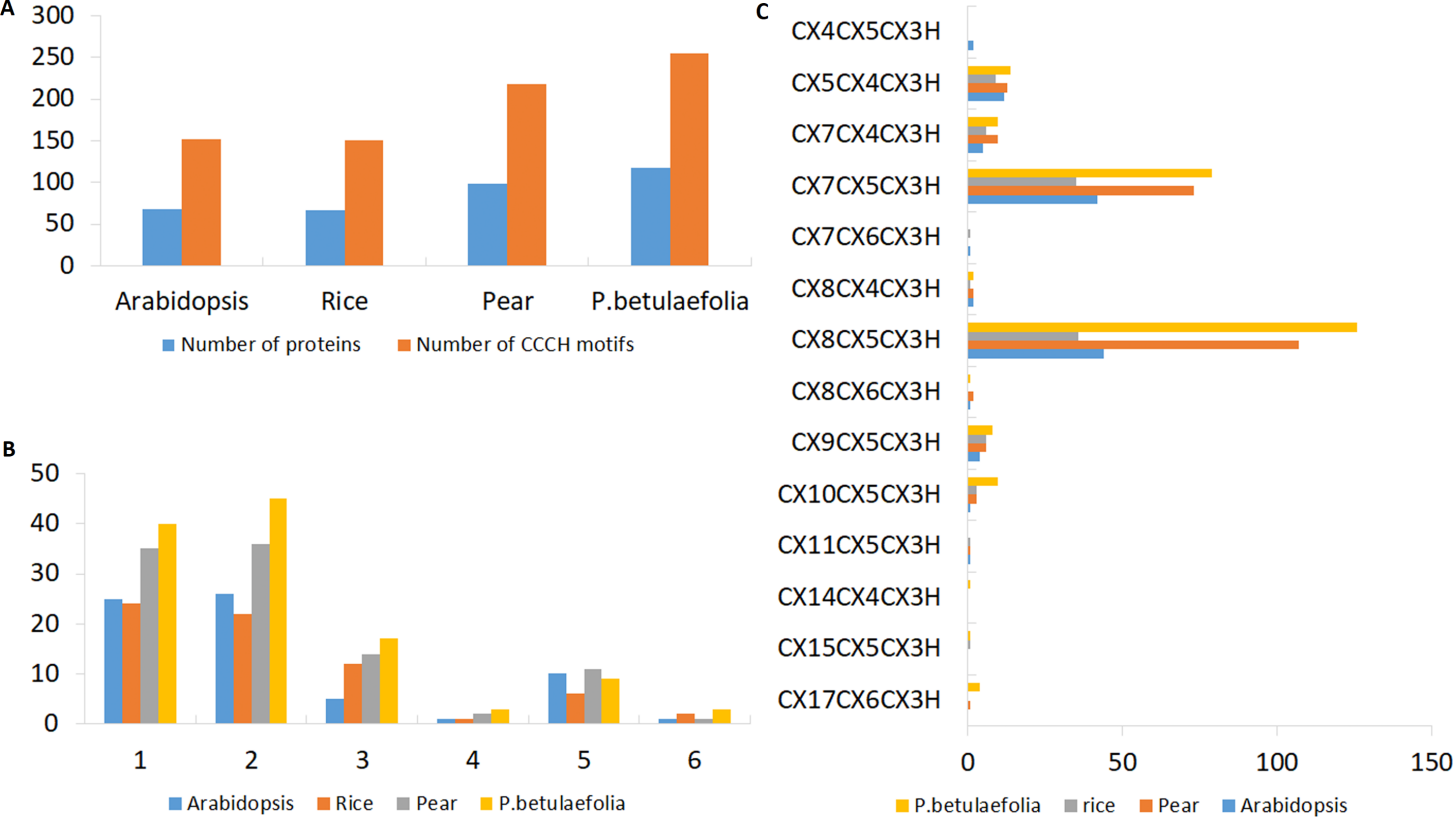
Characterizations of the C3H Znf proteins. (A) Number of C3H motifs identified in pear, rice and *Arabidopsis*. (B) Different number of C3H motifs per protein in pear, rice and *Arabidopsis*. (C) Number of different types of C3H motifs in pear, rice and *Arabidopsis*.

The released whole-genome sequence of pear (Wu et al., 2013) and *P. betulaefolia* (Dong et al., 2019) was used in the present study. To identify C3H family genes in the genome sequence dataset, we performed a Hidden Markov Model (HMM) search using the C3H domain file (PF00642) as a query and 120 and 124 sequences were identified in *P. betulaefolia* and pear, respectively. After HMM search and manual analysis to remove false positive and redundant genes, a total of 117 and 99 non-redundant, full-length *C3H* genes in *P. betulaefolia* and pear were identified and designated *PbeC3H1-PbeC3H117* (Table 1 and Table S2). Based on the different amino-acid spacing numbers between Cys and His in Znf motif, we found 11 types but excluded three types that contained zero C3H members: (C-X_4_-C-X_5_-C-X_3_-H, C-X_7_-C-X_6_-C-X_3_-H and C-X_15_-C-X_5_-C-X_3_-H) (Fig. 1). Characterizations of the 117 PbeC3H proteins, including number of amino acids (length), number of C3H motifs, molecular weight (MW), isoelectric point (pI) and physical location are listed in Table 1. We found that the deduced full lengths of PbeC3H proteins ranged from 142 (PbeC3H72) to 2040 amino acids (aa) (PbeC3H110) with an average of 613 aa, among which only 12 of the C3H genes were more than 1000 aa in length. The relevant MW were 16.03 kDa for PbeC3H72 and 223.61 kDa for PbeC3H110. The pI ranged from 4.72 (PbeC3H101) to 9.69 (PbeC3H102).

**Table 1:** The characteristics of C3H family members in *P. betulaefolia*.

**Gene**	**protein ID**	**Chr.**	**Position**	**Number of CCCH**	**No. of Intron**	**pI**	**Mw (kDa)**	**Length of AA**
PbeC3H1	GWHPAAYT004872	Chr10	GWHAAYT00000010:25730504-25736321( +)	6	11	7.79	54.22	499
PbeC3H2	GWHPAAYT026042	Chr16	GWHAAYT00000016:10835842-10838359( +)	5	6	8.66	51.79	483
PbeC3H3	GWHPAAYT014629	Chr13	GWHAAYT00000013:11223808-11226654( +)	5	6	8.71	50.24	472
PbeC3H4	GWHPAAYT054422	Chr9	GWHAAYT00000009:6818366-6822407( +)	5	6	8.51	50.25	462
PbeC3H5	GWHPAAYT046338	Chr6	GWHAAYT00000006:19036165-19050554( -)	6	21	5.41	122.09	1089
PbeC3H6	GWHPAAYT012885	Chr12	GWHAAYT00000012:27536359-27539624( -)	5	6	8.84	48.30	443
PbeC3H7	GWHPAAYT028883	Chr17	GWHAAYT00000017:7152160-7156121( +)	5	6	8.52	49.90	461
PbeC3H8	GWHPAAYT009701	Chr11	GWHAAYT00000011:34084040-34086781( -)	5	6	9.08	50.76	472
PbeC3H9	GWHPAAYT037374	Chr3	GWHAAYT00000003:30260879-30269328( -)	5	12	6.74	90.17	827
PbeC3H10	GWHPAAYT018571	Chr14	GWHAAYT00000014:19759504-19762970( -)	5	5	4.85	69.15	627
PbeC3H11	GWHPAAYT018570	Chr14	GWHAAYT00000014:19749693-19758430( -)	6	13	5.28	119.84	1077
PbeC3H12	GWHPAAYT018569	Chr14	GWHAAYT00000014:19736980-19742852( -)	4	5	7.84	61.27	548
PbeC3H13	GWHPAAYT031393	Chr2	GWHAAYT00000002:2031754-2033292( -)	3	1	6.60	38.30	340
PbeC3H14	GWHPAAYT021136	Chr15	GWHAAYT00000015:11096960-11098700( -)	3	1	8.27	38.21	340
PbeC3H15	GWHPAAYT044803	Chr6	GWHAAYT00000006:3732359-3736041( -)	3	1	7.12	37.83	344
PbeC3H16	GWHPAAYT027356	Chr16	GWHAAYT00000016:23467664-23473889( +)	3	2	6.59	83.27	751
PbeC3H17	GWHPAAYT046335	Chr6	GWHAAYT00000006:19016194-19020473( -)	3	5	5.42	51.70	451
PbeC3H18	GWHPAAYT040308	Chr4	GWHAAYT00000004:27566275-27567502( -)	2	2	6.16	26.99	247
PbeC3H19	GWHPAAYT017142	Chr14	GWHAAYT00000014:5501838-5504673( -)	3	2	9.42	30.09	288
PbeC3H20	GWHPAAYT056173	Chr9	GWHAAYT00000009:23753571-23754619( +)	2	1	6.46	34.89	309
PbeC3H21	GWHPAAYT010413	Chr12	GWHAAYT00000012:5269776-5272693( -)	3	2	9.37	31.47	302
PbeC3H22	GWHPAAYT006210	Chr11	GWHAAYT00000011:1040419-1043545( -)	3	2	9.32	31.98	302
PbeC3H23	GWHPAAYT030661	Chr17	GWHAAYT00000017:26070704-26071724( +)	2	1	7.13	33.68	299
PbeC3H24	GWHPAAYT034368	Chr3	GWHAAYT00000003:1946353-1948269( -)	2	1	9.00	27.51	258
PbeC3H25	GWHPAAYT024734	Chr16	GWHAAYT00000016:1951119-1952453( -)	2	1	6.60	37.41	332
PbeC3H26	GWHPAAYT018566	Chr14	GWHAAYT00000014:19711176-19722925( -)	3	20	5.52	97.80	869
PbeC3H27	GWHPAAYT013276	Chr13	GWHAAYT00000013:1972178-1976710( -)	2	5	6.46	70.32	622
PbeC3H28	GWHPAAYT034077	Chr2	GWHAAYT00000002:26038459-26043806( -)	4	7	7.71	45.80	411
PbeC3H29	GWHPAAYT000077	Chr1	GWHAAYT00000001:928279-930632( +)	3	1	8.77	82.70	740
PbeC3H30	GWHPAAYT047287	Chr7	GWHAAYT00000007:641667-646360( -)	4	6	7.94	40.78	368
PbeC3H31	GWHPAAYT007652	Chr11	GWHAAYT00000011:14107273-14109742( +)	2	4	9.52	36.70	310
PbeC3H32	GWHPAAYT057935	Scaffold13	GWHAAYT00000030:524170-526802( -)	2	4	9.54	38.05	325
PbeC3H33	GWHPAAYT016608	Chr14	GWHAAYT00000014:1217832-1220347( +)	2	4	9.48	37.96	325
PbeC3H34	GWHPAAYT037439	Chr3	GWHAAYT00000003:30730655-30736825( +)	2	13	6.15	114.74	1015
PbeC3H35	GWHPAAYT044398	Chr6	GWHAAYT00000006:164404-169644( +)	2	11	9.09	80.95	692
PbeC3H36	GWHPAAYT035367	Chr3	GWHAAYT00000003:10446769-10454852( -)	2	11	8.29	107.94	937
PbeC3H37	GWHPAAYT035368	Chr3	GWHAAYT00000003:10461664-10469805( -)	2	12	6.68	111.28	967
PbeC3H38	GWHPAAYT035458	Chr3	GWHAAYT00000003:11484892-11493030( +)	2	12	6.68	111.28	967
PbeC3H39	GWHPAAYT035459	Chr3	GWHAAYT00000003:11499894-11507981( +)	2	11	7.99	114.44	992
PbeC3H40	GWHPAAYT043462	Chr5	GWHAAYT00000005:31128036-31134192( +)	2	8	6.08	73.74	633
PbeC3H41	GWHPAAYT030146	Chr17	GWHAAYT00000017:21761540-21763264( +)	2	1	9.21	30.19	269
PbeC3H42	GWHPAAYT024365	Chr15	GWHAAYT00000015:45055282-45065570( +)	2	14	7.86	143.22	1242
PbeC3H43	GWHPAAYT049001	Chr7	GWHAAYT00000007:16963722-16964921( -)	1	3	8.16	34.60	304
PbeC3H44	GWHPAAYT057319	Contig7	GWHAAYT00000054:142463-143662( +)	1	3	8.34	35.14	308
PbeC3H45	GWHPAAYT000810	Chr1	GWHAAYT00000001:8333661-8334865( -)	1	2	6.06	35.94	316
PbeC3H46	GWHPAAYT021404	Chr15	GWHAAYT00000015:13293137-13297144( +)	3	6	6.11	107.69	984
PbeC3H47	GWHPAAYT039624	Chr4	GWHAAYT00000004:22915022-22916168( +)	1	2	6.24	35.70	317
PbeC3H48	GWHPAAYT037541	Chr4	GWHAAYT00000004:20463-25338( -)	1	9	8.32	42.20	390
PbeC3H49	GWHPAAYT031715	Chr2	GWHAAYT00000002:4399000-4402315( -)	2	9	6.20	67.20	606
PbeC3H50	GWHPAAYT058631	Scaffold24	GWHAAYT00000041:131755-134103( -)	2	3	9.16	36.49	325
PbeC3H51	GWHPAAYT021385	Chr15	GWHAAYT00000015:13170843-13174257( -)	2	9	6.42	67.54	606
PbeC3H52	GWHPAAYT030139	Chr17	GWHAAYT00000017:21665180-21666657( +)	2	1	9.15	30.26	270
PbeC3H53	GWHPAAYT046336	Chr6	GWHAAYT00000006:19022556-19029581( -)	1	10	8.33	48.64	425
PbeC3H54	GWHPAAYT022983	Chr15	GWHAAYT00000015:28484438-28489111( -)	3	2	9.15	99.03	885
PbeC3H55	GWHPAAYT051284	Chr8	GWHAAYT00000008:4635900-4636979( -)	2	0	9.09	39.61	359
PbeC3H56	GWHPAAYT030480	Chr17	GWHAAYT00000017:24593950-24595263( -)	2	0	8.05	47.78	437
PbeC3H57	GWHPAAYT006773	Chr11	GWHAAYT00000011:6308703-6310411( +)	1	2	8.74	49.80	422
PbeC3H58	GWHPAAYT034798	Chr3	GWHAAYT00000003:5499738-5501559( +)	1	2	8.64	54.84	468
PbeC3H59	GWHPAAYT001887	Chr1	GWHAAYT00000001:15813371-15816672( -)	2	7	7.14	42.01	387
PbeC3H60	GWHPAAYT005485	Chr10	GWHAAYT00000010:30174061-30177245( +)	1	4	5.12	85.24	770
PbeC3H61	GWHPAAYT050090	Chr7	GWHAAYT00000007:25191629-25195132( -)	2	8	7.14	47.22	434
PbeC3H62	GWHPAAYT056049	Chr9	GWHAAYT00000009:22592310-22593644( -)	2	0	6.67	48.90	444
PbeC3H63	GWHPAAYT049908	Chr7	GWHAAYT00000007:23993782-23994672( +)	2	0	7.89	34.33	296
PbeC3H64	GWHPAAYT031639	Chr2	GWHAAYT00000002:3718563-3719777( +)	2	0	6.56	44.28	404
PbeC3H65	GWHPAAYT020039	Chr15	GWHAAYT00000015:3758094-3759338( -)	2	1	6.63	42.70	386
PbeC3H66	GWHPAAYT043888	Chr5	GWHAAYT00000005:34068173-34071488( +)	1	4	5.34	90.44	814
PbeC3H67	GWHPAAYT007452	Chr11	GWHAAYT00000011:11989508-11994851( +)	1	14	5.50	90.89	833
PbeC3H68	GWHPAAYT035218	Chr3	GWHAAYT00000003:8805509-8810690( +)	1	13	5.48	90.74	830
PbeC3H69	GWHPAAYT021328	Chr15	GWHAAYT00000015:12834928-12836121( +)	2	0	6.61	43.58	397
PbeC3H70	GWHPAAYT033934	Chr2	GWHAAYT00000002:24630931-24633862( -)	1	7	5.96	77.20	719
PbeC3H71	GWHPAAYT024373	Chr15	GWHAAYT00000015:45121249-45132331( +)	1	14	6.94	146.29	1271
PbeC3H72	GWHPAAYT012207	Chr12	GWHAAYT00000012:23241819-23242865( +)	1	3	7.69	16.03	142
PbeC3H73	GWHPAAYT008395	Chr11	GWHAAYT00000011:23033685-23037002( -)	1	7	6.27	71.50	642
PbeC3H74	GWHPAAYT036140	Chr3	GWHAAYT00000003:19611823-19615676( -)	1	6	5.98	75.18	685
PbeC3H75	GWHPAAYT008394	Chr11	GWHAAYT00000011:23001654-23005914( -)	1	6	5.77	75.91	693
PbeC3H76	GWHPAAYT021131	Chr15	GWHAAYT00000015:11077946-11079958( -)	3	2	4.93	43.53	392
PbeC3H77	GWHPAAYT056600	Contig11	GWHAAYT00000058:4512-6882( +)	2	6	9.03	49.63	457
PbeC3H78	GWHPAAYT036141	Chr3	GWHAAYT00000003:19631658-19634938( -)	1	7	6.29	69.73	625
PbeC3H79	GWHPAAYT032958	Chr2	GWHAAYT00000002:14472402-14474766( -)	2	5	9.01	53.33	487
PbeC3H80	GWHPAAYT036362	Chr3	GWHAAYT00000003:22251997-22254706( +)	1	6	6.43	63.04	557
PbeC3H81	GWHPAAYT008775	Chr11	GWHAAYT00000011:26571528-26574945( +)	2	4	6.03	55.53	507
PbeC3H82	GWHPAAYT049718	Chr7	GWHAAYT00000007:22823019-22825010( +)	2	0	7.51	72.74	663
PbeC3H83	GWHPAAYT017283	Chr14	GWHAAYT00000014:6682374-6684572( -)	2	0	6.05	79.63	732
PbeC3H84	GWHPAAYT058639	Scaffold25	GWHAAYT00000042:5861-8059( -)	2	0	6.05	79.63	732
PbeC3H85	GWHPAAYT010500	Chr12	GWHAAYT00000012:6337374-6339584( -)	2	0	5.94	80.05	736
PbeC3H86	GWHPAAYT024495	Chr16	GWHAAYT00000016:503060-505966( +)	3	2	8.67	72.79	652
PbeC3H87	GWHPAAYT006735	Chr11	GWHAAYT00000011:6089514-6091628( -)	2	0	5.70	76.88	704
PbeC3H88	GWHPAAYT034763	Chr3	GWHAAYT00000003:5237873-5239969( -)	2	0	5.80	76.34	698
PbeC3H89	GWHPAAYT013030	Chr13	GWHAAYT00000013:529675-532829( +)	3	2	5.56	82.48	741
PbeC3H90	GWHPAAYT032233	Chr2	GWHAAYT00000002:7924881-7931140( +)	1	9	5.75	155.91	1417
PbeC3H91	GWHPAAYT005126	Chr10	GWHAAYT00000010:27598083-27603908( +)	3	6	7.23	73.25	669
PbeC3H92	GWHPAAYT043550	Chr5	GWHAAYT00000005:31745087-31750861( +)	3	6	6.38	73.49	671
PbeC3H93	GWHPAAYT002960	Chr10	GWHAAYT00000010:5349432-5351904( +)	1	2	8.41	80.44	755
PbeC3H94	GWHPAAYT019605	Chr15	GWHAAYT00000015:730010-733989( -)	1	3	6.09	46.52	428
PbeC3H95	GWHPAAYT001497	Chr1	GWHAAYT00000001:13445177-13449529( +)	2	2	8.55	80.82	735
PbeC3H96	GWHPAAYT011456	Chr12	GWHAAYT00000012:17323959-17326963( -)	2	7	6.98	40.72	359
PbeC3H97	GWHPAAYT039053	Chr4	GWHAAYT00000004:18005521-18008144( -)	2	7	5.41	43.07	376
PbeC3H98	GWHPAAYT032215	Chr2	GWHAAYT00000002:7795622-7799068( -)	1	3	9.25	54.07	497
PbeC3H99	GWHPAAYT021769	Chr15	GWHAAYT00000015:15920198-15923173( -)	1	3	9.35	53.12	491
PbeC3H100	GWHPAAYT049365	Chr7	GWHAAYT00000007:20269487-20273825( -)	2	6	5.36	44.17	388
PbeC3H101	GWHPAAYT041019	Chr5	GWHAAYT00000005:6231421-6243045( +)	1	9	4.72	182.06	1686
PbeC3H102	GWHPAAYT055054	Chr9	GWHAAYT00000009:11732453-11735229( +)	1	8	9.69	56.89	502
PbeC3H103	GWHPAAYT054501	Chr9	GWHAAYT00000009:7330066-7333040( +)	1	6	6.50	64.03	562
PbeC3H104	GWHPAAYT001179	Chr1	GWHAAYT00000001:11358181-11362387( -)	2	7	5.19	41.36	366
PbeC3H105	GWHPAAYT049306	Chr7	GWHAAYT00000007:19777362-19779399( -)	1	2	8.33	55.43	498
PbeC3H106	GWHPAAYT056822	Contig23	GWHAAYT00000070:61051-63087( -)	1	2	8.33	55.41	498
PbeC3H107	GWHPAAYT028963	Chr17	GWHAAYT00000017:7706609-7709177( +)	1	6	5.91	63.69	562
PbeC3H108	GWHPAAYT051081	Chr8	GWHAAYT00000008:3319927-3322376( -)	1	3	5.14	55.38	501
PbeC3H109	GWHPAAYT053823	Chr9	GWHAAYT00000009:2857541-2864019( +)	1	13	6.38	127.67	1153
PbeC3H110	GWHPAAYT020685	Chr15	GWHAAYT00000015:8141321-8154629( -)	5	9	8.83	223.61	2040
PbeC3H111	GWHPAAYT054318	Chr9	GWHAAYT00000009:6055665-6060630( -)	1	11	9.10	45.61	403
PbeC3H112	GWHPAAYT024476	Chr16	GWHAAYT00000016:394457-396533( +)	1	2	6.19	52.86	464
PbeC3H113	GWHPAAYT021795	Chr15	GWHAAYT00000015:16131190-16137379( +)	1	9	5.76	158.43	1441
PbeC3H114	GWHPAAYT053636	Chr9	GWHAAYT00000009:1667637-1676699( -)	1	10	6.15	159.11	1468
PbeC3H115	GWHPAAYT028069	Chr17	GWHAAYT00000017:1526738-1536447( -)	1	6	6.13	163.16	1505
PbeC3H116	GWHPAAYT019049	Chr14	GWHAAYT00000014:22751706-22755724( -)	1	3	9.05	107.92	974
PbeC3H117	GWHPAAYT008452	Chr11	GWHAAYT00000011:23501475-23502838( +)	1	1	9.26	47.21	422

**Figure 2:**
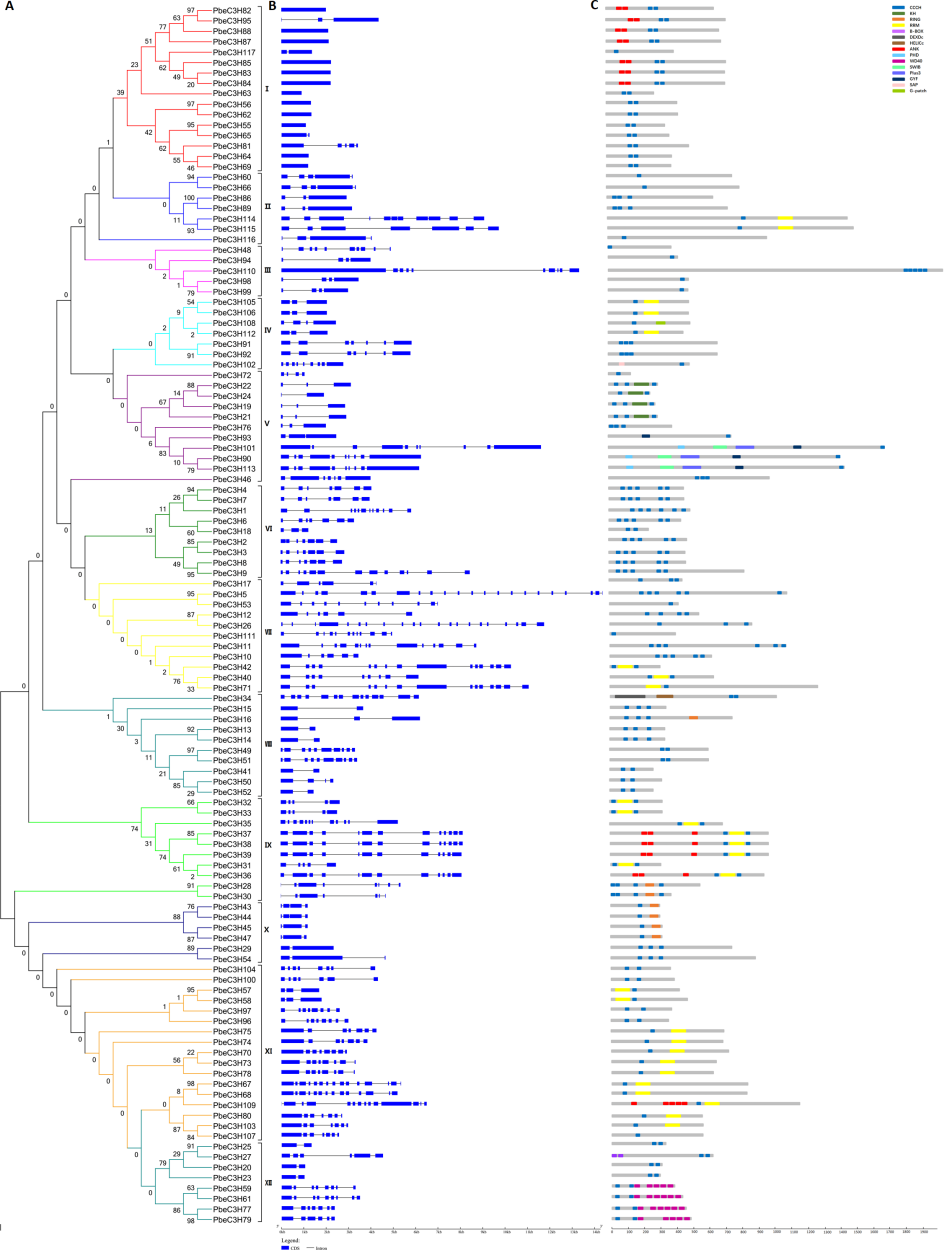
Phylogenetic and structural analysis of the *PbC3H* family genes. (A) Phylogenetic tree of the *C3H* family in pear was generated using the neighbor-joining with 1,000 bootstraps in MEGA 7. (B) Gene structure of the intron-exon and (C) motifs in each C3H proteins.

There were more C3H genes found in *P. betulaefolia* (117) than that in pear (99) and reported for *Arabidopsis* (68) and rice (67) (Fig. 1A). The numbers of C3H motifs varied accordingly in the three plants (Fig. 1B). the on line tool SMART, MOTIF Search and Pfam databases were used to calculate the total number of C3H Znf motifs in the PbeC3H proteins, and a total of 255 C3H Znf motifs were identified, which exceeded those found in pear (218) (Table S2), *Arabidopsis* (152) and rice (150) (Wang et al., 2008). We found 1-6 C3H type domains in members of the pear C3H family, and some C3H proteins also carried several other known functional domains, including ANK, KH, RRM, SAP, WD-40, B-box, DEXDc, HELICc, PHD, SWIB, Plus3, GYF, G-patch and ZF-Ring (Fig. 2C), consistent with previous studies (Hudson et al., 2004; Wang et al., 2008; Kramer, Kimblin & Carrington, 2010). We found that the majority of members had either one (40 members) or two (45 members) C3H domains, representing 72.6% of the 117 *PbeC3H* genes. However, nine members contained five C3H domains, three contained four and three contained six, and 17 contained three domains and one contained one domain (Table 1). As similar results for *P. betulaefolia*, *Arabidopsis* and rice, the most common types of C3H motifs in *P. betulaefolia* were C-X_8_-C-X_5_-C-X_3_-H (49.4%) and C-X_8_-C-X_5_-C-X_3_-H (31.0%) (Fig. 1C). The motif C-X_15_-C-X_5_-C-X_3_-H was only found in member PbeC3H102, motif C-X_8_-C-X_6_-C-X_3_-H was only found in member PbeC3H110 and motif C-X_14_-C-X_6_-C-X_3_-H was only found in PbeC3H117 (Fig. 1C).

### Phylogeny, classification and structural organization of *PbeC3H* genes

We constructed a Maximum Likelihood tree based on alignment of the full-length amino acid sequences of the 117 PbeC3Hs to illustrate the evolutionary relationships between them (Fig. 2A). Based on the relationships or clades between proteins and the protein structures or motifs, the 117 PbeC3Hs were divided into 12 groups, designated I-XII. The PbeC3Hs in the same clade shared similar exon-intron structures of their encoding sequences (Fig. 2B) and similar numbers and distributions of functional motifs (Fig. 2C). Conserved exon-intron structures and motif types and number distribution across the PbeC3Hs in each clade strongly supported the reliability of the phylogenetic tree. There were 16 PbeC3H members in group I, seven in group II, five in group III, seven in group IV, 11 in group V, nine in group VI, 11 in group VII, ten in group VIII, ten in group IX, six in group X, 17 in group XI and eight in group XII (Fig. 2A). Group XI and I were the first and second largest with 17 and 16 members. However, group I showed the simplest exon-intron structures. Most of them (12 members) had no intron; *PbeC3H65*, *PbeC3H95* and *PbeC3H117* had one or two introns while *PbeC3H81* showed much more complex gene structures (Figs. 2A, 2B). Except for most members in group X and some in groups VIII and XII, which contained one or two intron-(s), members of the other groups contained 3-21 introns. Interestingly, although exon-intron organization of C3H genes varied considerably in terms of intron numbers, the intron phase was remarkably well conserved, indicative of exon shuffling during evolution (Kolkman & Stemmer, 2001).

We also noted that the majority of the phylogenetic clades had well-supported bootstrap values, but bootstrap values of some proteins were low at the nodes and the phylogenetic relationships were unclear (Fig. 2A). Even so, considered together with exon-intron structures and conserved motifs, we could also perform gene classification and further analysis. Functional and divers motifs were found among PbeC3Hs including RRMs and K homolog domains (KH) that are involved in RNA processing, and Ankyrin repeats (Ank), WD40 repeats (WD40) and ZF-Ring motifs that are involved in protein-protein interactions or multi-protein complex assembly (Fig. 2C). The conserved motifs were one important basis for classification of C3H genes. For example, the ANK motif was only found in group I, IX and XI, RRM was relatively found in PbeC3H members, the WD-40 was only found in group XII, ZF-Ring was found in groups IX and X, the KH and SWIB motifs were only found in groups V. There are some other motifs such as SAP, B-BOX, DEXDc, HELICc, PHD and G-patch were found in one or two member(s) and Moreover, RRM and KH domain-containing proteins have been demonstrated to play essential roles in many aspects of RNA metabolism, suggesting that the *P. betulaefolia* C3H proteins harboring these domains may function as RNA-binding proteins and are involved in RNA processing. For example, members PbeC3H19, PbeC3H21, PbeC3H22 and PbeC3H24 contained the conserved KH domain, suggesting that this domain plays important subfamily-specific functions. The phylogenetic reconstruction was further supported by analysis of domain architecture.

### Chromosomal locations of *PbeC3H* genes

**Figure 3:**
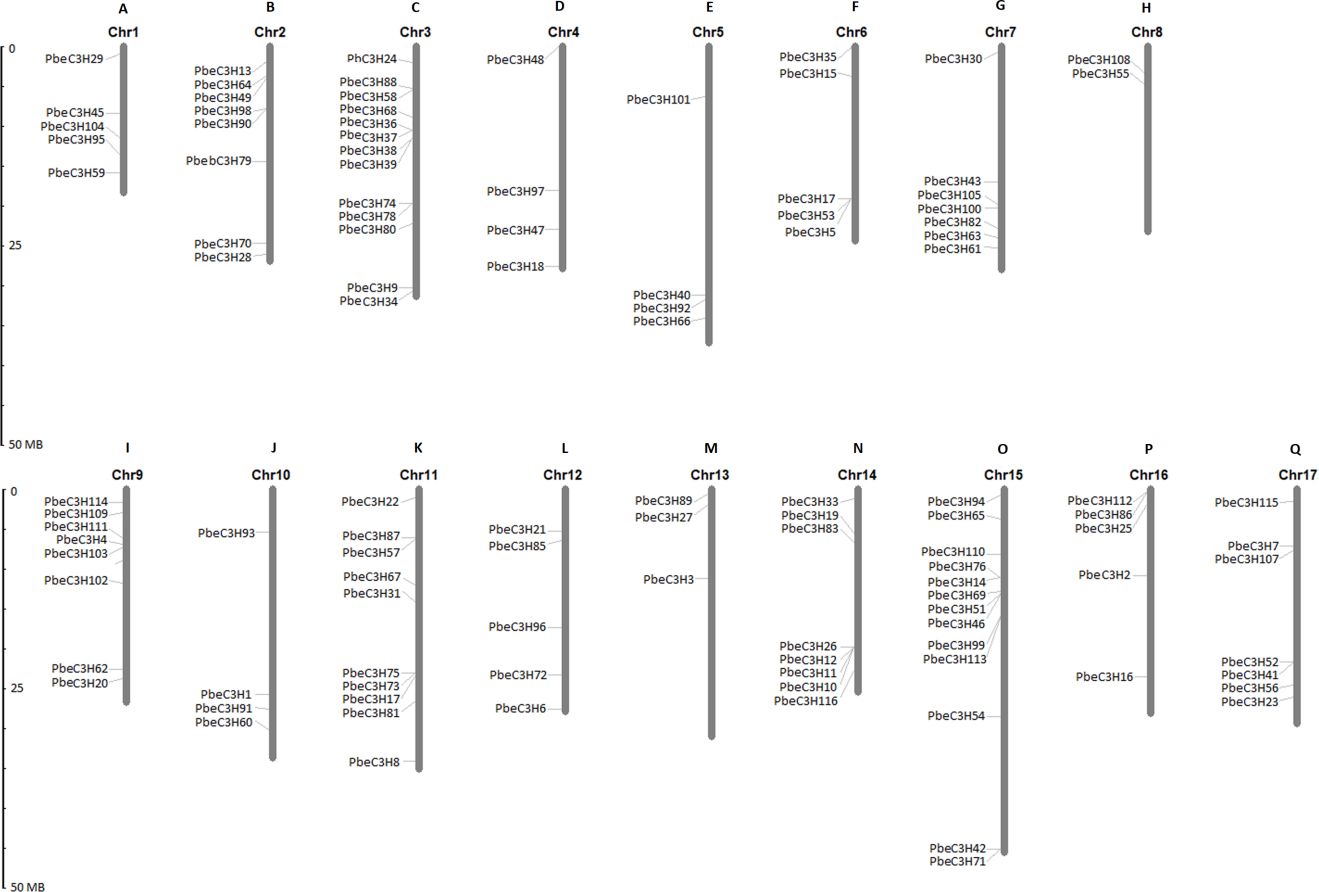
Chromosomal distributions of *C3H* genes. The Roman numerals on top of each chromosome represent the number of the chromosome. (A) Chr1, (B) Chr2, (C) Chr3, (D) Chr4, (E) Chr5, (F) Chr6, (G) Chr7, (H) Chr8, (I) Chr9, (J) Chr10, (K) Chr11, (L) Chr12, (M) Chr13, (N) Chr14, (O) Chr15, (P) Chr16 (Q) Chr17.

Based on the starting position of each gene on the chromosomes, 111 of the 117 *PbeC3H* genes were physically located on 17 chromosomes, and 6 genes (*PbeC3H32*, *PbeC3H44*, *PbeC3H50*, *PbeC3H77*, *PbeC3H84* and *PbeC3H106*) remained on unattributed scaffold or contig fragments (Fig. 3 and Table 1). The distribution of *PbeC3H* genes among chromosomes appeared to be uneven: chromosomes 4, 5, 8, 10 and 13 harbored two to four C3H genes, and relatively high densities of C3Hs were discovered on chromosomes 2, 3, 9, 10, 11 and 15 with more than eight C3H genes. Chromosomes 3 and 15 contained the largest number of C3H genes (13 each) followed by chromosome 11 (ten) and chromosome 2, 9 and 11 (eight each), and sevsn each on chromosome 7 and 17. Notably, some C3Hs located on chromosomes 3, 6, 9, 11, 14 and 15 were arranged in clusters (Fig. 3).

### Evolutionary clues of C3H genes in *P. betulaefolia* and apple

**Figure 4:**
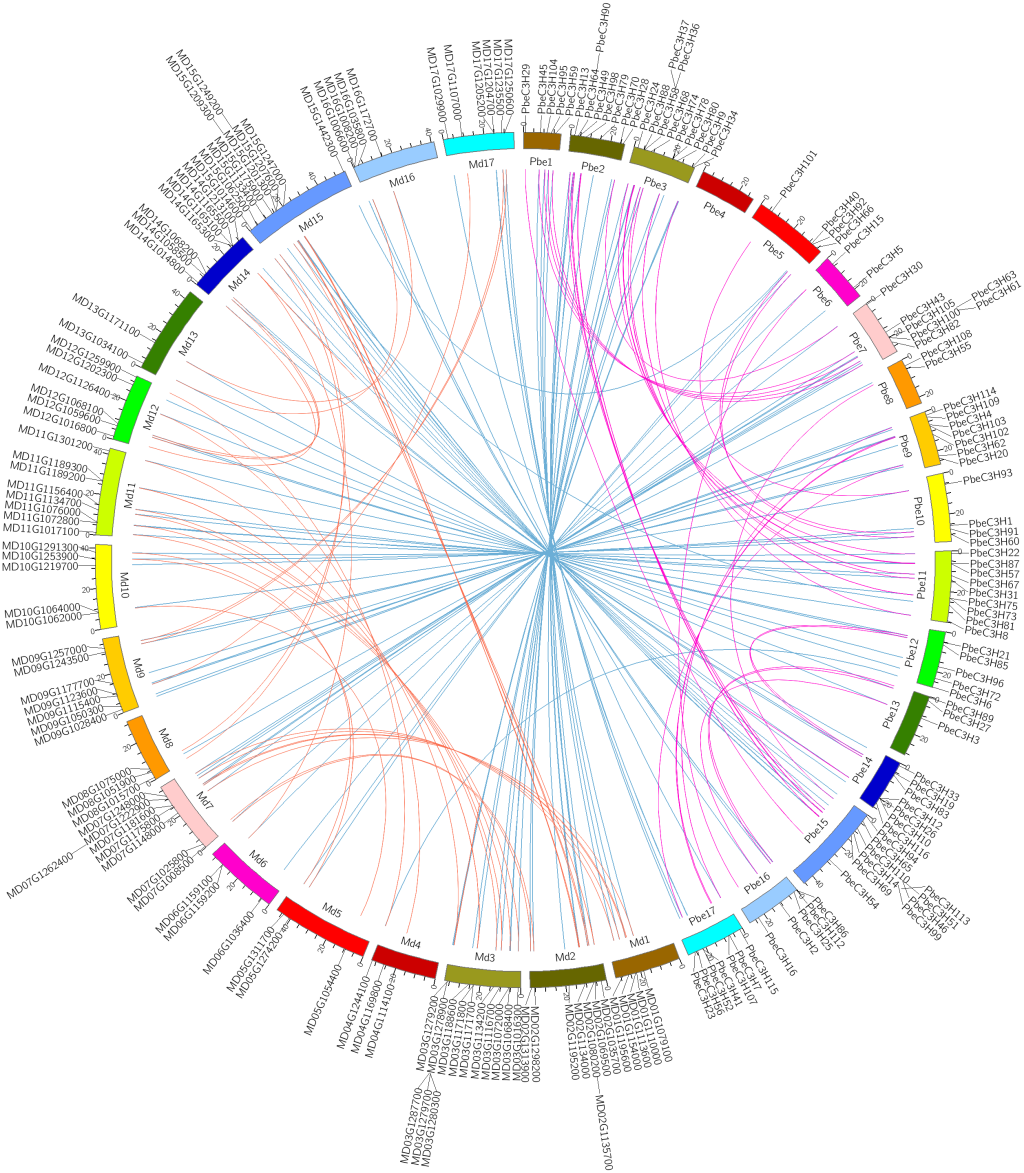
Intra- and interspecific comparisons of *C3H* genes in pear and apple. All the *C3H* gene pairs are depicted in the pear chromosomes. The pink and orange lines indicate intraspecific synteny of *C3H* genes in pear and apple, the blue lines indicated interspecific synteny between pear and apple.

A recent whole-genome duplication (WGD) event shared by *P. betulaefolia* and apple occurred ∼50 million years ago (MYA), prior to divergence of the two groups ∼22.4–29.4 MYA, but after their divergence from strawberry (Wu et al., 2013; Daccord et al., 2017; Dong et al., 2019). Analysis of the relationship between C3H homologous gene pairs across *P. betulaefolia* and apple could provide insights into their divergence and evolution. A comparative analysis of the homologous C3H gene pairs across *P. betulaefolia* and apple was conducted (Fig. 4). Results showed 87 orthologous C3H gene pairs between them, and 35 and 37 paralogous pairs in *P. betulaefolia* and apple, respectively (Table S3). All the C3H members with synteny relationships were showed using Circos (Fig. 4) (Krzywinski et al., 2009). The *K*s values were used to estimate divergence time, which was in range of 0.012–0.125 for orthologous genes and 0.65–6.74 MYA for the time. The estimate of the divergence time was considerably less than that of the speciation time (22.4–39.4 MYA) (Dong et al., 2019). This suggested divergence of the orthologous gene pairs between *P. betulaefolia* and apple occurred after their speciation (Daccord et al., 2017; Dong et al., 2019). Moreover, the estimated divergence time based on *K*s values of the C3H paralogous gene within *P. betulaefolia* or apple genome was in the range of 5.77–19.40 and 5.45–15.76 MYA, respectively; both occurred after the WGD event in their common ancestor (Fig. 4 and Table S3). This indicates that the C3H genes in both *P. betulaefolia* and apple experienced the WGD event (Daccord et al., 2017; Dong et al., 2019).

Moreover, the selection types and divergence dates of duplicated gene pairs were investigated by calculating the synonymous (*K*s) and non-synonymous substitutions (*K*a) per site between duplicated pairs; *K*a/*K*s = 1 indicates neutral selection, *K*a/*K*s <1 indicates purifying selection, and *K*a/*K*s >1 indicates accelerated evolution with positive selection (Yang & Bielawski, 2000; Zhang et al., 2006). The *K*s, *K*a and *K*a/*K*s of 35 paralogous gene pairs of C3Hs in *P. betulaefolia* (Table S3) showed that all paralogous gene pairs of the C3H family had *K*s of 0.107–0.359 and *K*a/*K*s of 0.031–0.586 suggesting all 35 paralogous gene pairs of the PbeC3H family had undergone purifying selection during WGD. The *K*s values of 37 paralogous gene pairs of C3Hs in apple within 0.101–0.292 and *K*a/*K*s ratios was 0–0.877, indicating that they also had undergone purifying selection. The 87 orthologous C3H gene pairs between *P. betulaefolia* and apple showed slightly different type. Their *K*a/*K*s ratios was 0–2.198 indicating that most of them in the different species had undergone purifying selection. However, the *K*a/*K*s of one orthologous gene pairs in *P. betulaefolia* and apple, PbeC3H66 and MD05G1311700 (*K*a/*K*s = 20198) undergone strong positive selection (Table S3). The divergence time of the intra-genomic C3H genes was more than that of inter-genomic comparison between *P. betulaefolia* and apple. This is consistent with the WGD occurring before species differentiation of *P. betulaefolia* and apple (Daccord et al., 2017; Dong et al., 2019).

### Many *PbeC3H* genes showed induced expression under salt stress

**Figure 5:**
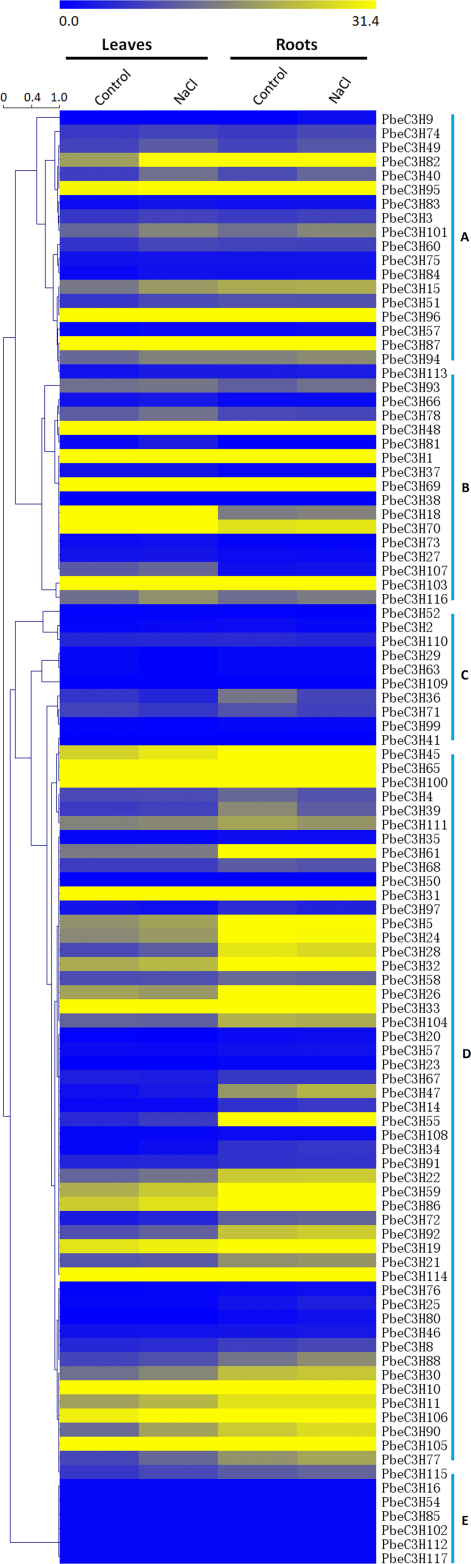
Expression patterns of *C3H* genes under salt stress in leaves and roots. Scale bars represent the RPKM values. The heat map was drawn using a single color gradient. Groups (A–E) were marked on the right of the picture.

To investigate the expression patterns of the *PbeC3H* genes, a comprehensive expression analysis was performed based on whole-genome coverage. We analyzed the expression patterns of *PbeC3H* genes under salt stress using RNA-Seq data generated in a previous study. The RPKM values were used as expressions (Li et al., 2017). Of the 117 C3H genes, 103 showed expression in at least one selected treatment in leaves or roots (Fig. 5). Results indicated that C3H genes showed varied expression patterns in leaves or roots under salt stress. Expression of many of the *PbeC3H* genes was obviously induced under salt stress in leaves and/or roots. We divided these into five groups according to expression patterns in different organs or under salt stress. To further elucidate the transcription patterns of C3H genes, their expression patterns were clustered across different groups. In general, different groups showed different expression patterns (Fig. 5), suggesting functional divergence of different members of C3H genes. For example, expression of some members in groups a, c and e (Figs. 5A, 5C and 5E) and some in group d (Fig. 5D) was hardly detected in leaves or roots for either salt or control. However, C3H members in groups b and d (Figs. 5B and 5D) and most in group f (Fig. 5F) were relatively highly expressed in all samples (Fig. 5).

Additionally, Gene Ontology (GO) (https://www.ebi.ac.uk/QuickGO/) analysis was performed to illustrate the function classification of the 117 C3H genes (Table S4). Results showed that the functions of most C3H genes were enriched in metal ion binding, mRNA binding, nuclease activity, transcription factor activity, sequence-specific DNA binding, mRNA 3′-UTR binding, transcription regulatory region DNA binding and transferase activity, transferase activity, etc. This was mostly consistent with the classification of the C3H genes in the phylogenetic tree or the heat map. Results indicate the C3H genes were involved in stress response, such as salt ions transport and metabolism.

**Figure 6:**
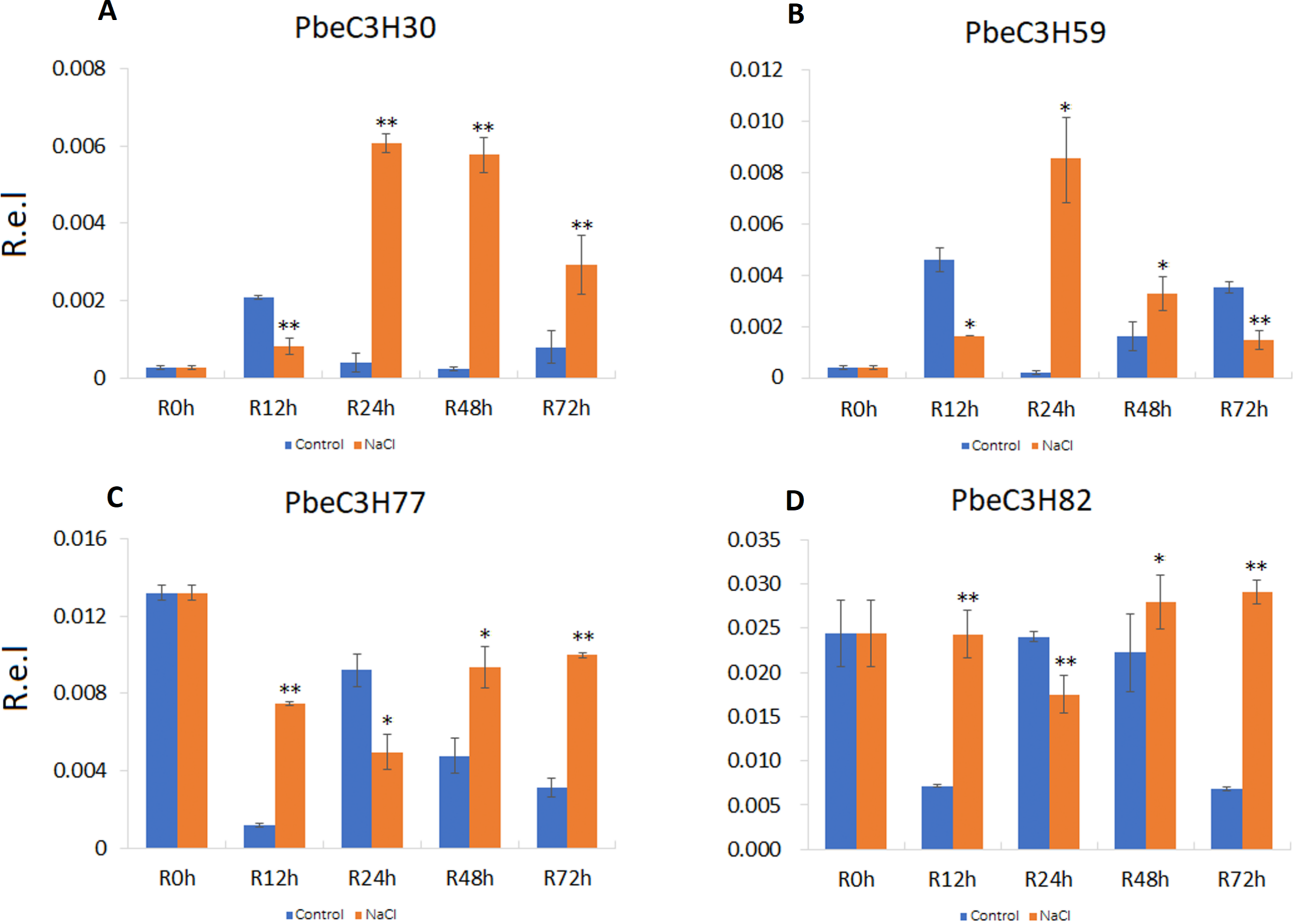
Expression profiles of *C3H* genes induced under salt stress treatment in roots. (A–D) Expression for each *PbeC3H* gene (A) PbeCSH30; (B) PbeC3H59; (C) PbeC3H77; (D) PbeC3H82. Relative expression was calculated using the 2^−ΔΔ*CT*^ method. R.e.l indicates relative expression level. The asterisk and double asterisks represent significant differences at the levels of 0.05 and 0.01, respectively.

To further analyze and validate their expression under salt stress, 18 *PbeC3H* genes were selected for quantitative real-time PCR. Results showed that four genes (*PbeC3H30*, *PbeC3H59*, *PbeC3H77* and *PbeC3H82*) were highly induced by salt stress treatment in roots (Fig. 6). Especially for PbeC3H30, expression was very greatly induced after 24 h under salt stress treatment. Gene *PbeC3H59* was induced at 24 and 48 h, but repressed at 12 and 72 h. The *PbeC3H77* and *PbeC3H82* showed similar expression profiles, being repressed at 24 h but highly induced at the other three time points (Fig. 6). These may indicate complexity in *P. betulaefolia* root response to salinity.

**Figure 7:**
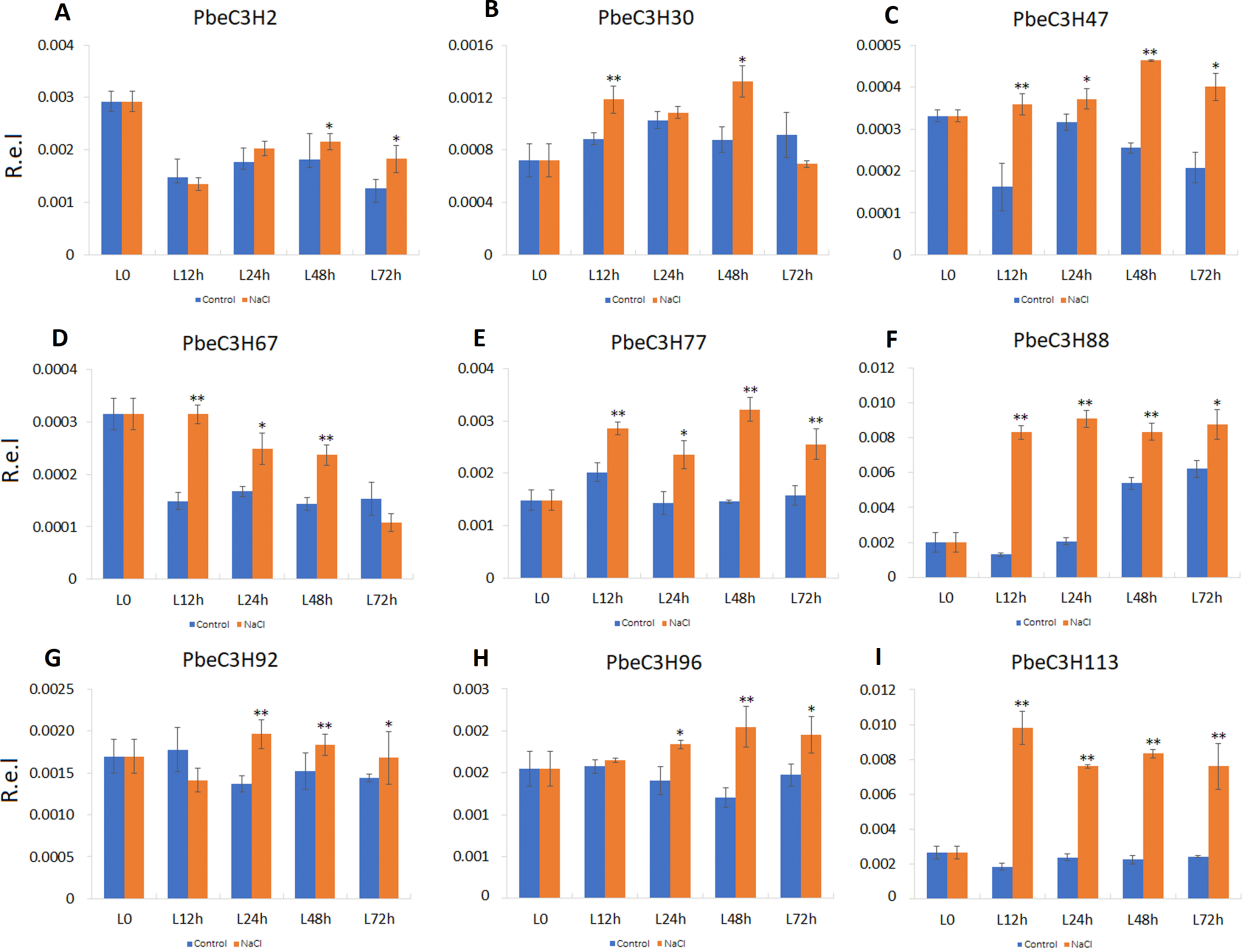
Expression patterns of *C3H* genes induced under salt stress treatment in leaves. (A–I) Expression for each *PbeC3H* gene ((A) PbeC3H2; (B) PbeC3H30; (C) PbeC3H47; (D) PbeC3H67; (E) PbeC3H77; (F) PbeC3H88; (G) PbeC3H92; (H) PbeC3H96; (I) PbeC3H113). Relative expression was calculated using the 2^−ΔΔ*CT*^ method. R.e.l indicates relative expression level. The asterisk and double asterisks represent significant differences at the levels of 0.05 and 0.01, respectively.

Compared to roots, *PbeC3H* genes in leaves showed a much more clearly response to salt. Nine of the selected genes (*PbeC3H2*, *PbeC3H30*, *PbeC3H47*, *PbeC3H67*, *PbeC3H96*, *PbeC3H77*, *PbeC3H88*, *PbeC3H92* and *PbeC3H113*) were significantly induced by salt at least at two time points (Fig. 7), consistent with the RNA-Seq data (Fig. 5). The *PbeC3H2* and *PbeC3H30* were only induced slightly at two time points. Except for *PbeC3H67*, *PbeC3H96* and *PbeC3H92* which were induced by salt at three time points, the other four genes, *PbeC3H47*, *PbeC3H77*, *PbeC3H88* and *PbeC3H113* and significantly induced expression at all the time points under salt stress treatment compared to controls. Notably, *PbeC3H113* showed expression levels of 4–5 times than those in controls (Fig. 7). These genes may play important roles in response to salinity and could be ideal candidates in improving salt tolerance in *P. betulaefolia*.

## Discussion

### C3H gene family in *P. betulaefolia*

Previous studies showed that the C3H proteins play important roles in many aspects of plant growth and development. Comparisons showed more C3H genes in *P. betulaefolia* than those reported in pear, *Arabidopsis* and rice (Fig. 1A and Table S2). The numbers of C3H motifs in the three plants varied accordingly, with *P. betulaefolia* containing the highest number of C3H motifs (256), followed by pear (218), Arabidopsis (152) and rice (150) (Fig. 1A). The MEME program was used to identify all motifs present in the C3H protein sequences. This led to prediction of a total of 15 different motifs including Znf-C3H (Fig. 2C). Similar to results for *Arabidopsis* and rice, the most common types of C3H motifs were C-X_8_-C-X_5_-C-X_3_-H followed by C-X_7_-C-X_5_-C-X_3_-H (Fig. 2C). In addition, some members contained unconventional C3H motifs. Although the C3H domain was highly conserved, the number of C3H domains and the spacing between adjacent C3H domains in a gene sequence and adjacent cysteines in the Znf motif in each gene were highly diverse (Wang et al., 2008). A previous study reported that ancestral genes containing the various C3H domain structures appeared early in evolution, and were maintained throughout evolution (Cai et al., 2013).

We also noted that the pI ranged from 4.72 (PbeC3H101) to 9.69 (PbeC3H101). It is because of the length of protein sequences were quite different between members and contained various motifs besides the basic C3H motif. The various values of pI may indicate different physicochemical property or three-dimensional structure to affect gene functions.

### Conserved gene structures may reveal specific functions

The C3H proteins have been found to regulate post-transcriptional modification of downstream target pre-mRNAs (Lai & Blackshear, 2001; Stefl, Skrisovska & Allain, 2005), interacting with different proteins (e.g., GhZFP1) (Guo et al., 2009) or transcriptionally activating/repressing target genes (e.g., *AtHUA1*, *AtPEI* and *OsLIC1*) (Li & Thomas, 1998; Li, Jia & Chen, 2001; Wang et al., 2008). The domain architecture and intron/exon structure of the C3H genes in *P. betulaefolia* were relatively complex but conserved within each group, and one of the bases to classify C3H members. The gene structure was generally consistent with the motif organization. For example, the complexity of structure of C3H members in group I and IX may be related to the number of ANK motifs (Fig. 2). The other examples, PbeC3H59, PbeC3H61, PbeC3H77 and PbeC3H79 in group XII which contained more than five WD-40 motifs each showed similar structure among them but different to the other members in this group. Similar situation in structure organization related to motifs was seen in PbeC3H43, PbeC3H44, PbeC3H45 and PbeC3H47, which contained one ZF-Rings each with different exon-intron structure (Fig. 2). These various but conserved gene structures and motif organizations may reveal functional divergence among different groups and provide excellent candidate genes for researching salt tolerance in *P. betulaefolia* breeding.

### *K*s of C3H genes in *P. beulaefolia* and apple provide evolutionary clues

The WGD is a major force in massive silencing and elimination of gene evolution (Jiao et al., 2011). Studies indicated that a WGD event occurred about 50 MYA in an ancestor of *P. betulaefolia* and apple, prior to divergence of these two taxa (Dong et al., 2019). In this study, we identified 117 *PbeC3H* genes in genomes, which had been subjected to the WGD event of the ancestor of *P. beulaefolia* and apple. Among them, there were 35 and 37 pairs of C3H genes in the *P. beulaefolia* and apple genome were paralogous, respectively (Table S3). An older divergence time was estimated for the paralogous gene pairs in *P. beulaefolia* and apple than for the orthologous gene pairs (Table S3). Results indicate the WGD event occurred prior to the speciation, consistent with the premise that the WGD occurred prior to divergence of *P. beulaefolia* and apple. Most of the C3H genes in *P. beulaefolia* and apple that were generated from the WGD event were retained, possibly due to their crucial roles in growth and development as well as response to environmental conditions. The relationship between homologous *PbeC3H* gene pairs will provide unique perspectives on evolution of the Rosaceae.

The paralogous C3H genes in *P. beulaefolia* and apple and most of those orthologous between themwith *K*a/*K*s<1 indicated purifying selection; however, *PbeC3H66* and *MD05G1311700* (*K*a/*K*s = 2.198) showed strong positive selection (Table S3). This indicated that *P. beulaefolia* and apple C3H orthologous genes had undergone significant selection after the species differentiation. The results in this paper provide excellent candidate genes to study the domestication of close relative species after their speciation.

### C3H genes expanded in *P. betulaefolia*

WGD or polyploidy, which results in massive silencing and elimination of duplicated genes, has long been recognized as a significant force in plant evolution (Jiao et al., 2011). In previous study, 17477 gene families were identified in *P. betulaefolia* lineage, among them, 2831 gene families were expanded in *P. betulaefolia*. The genes expanded are involved in stress and defence responses (Cui et al., 2016; Dong et al., 2019). In this study, we confirmed the results using C3H family genes. We saw C3H genes expanded obviously in *P. btulaefolia* (117) than that in *P. bretschneideri* (99) (Table S2), grape (69) (Wang, Zhong & Cheng, 2014), poplar (68) (Chai et al., 2012). The expansion of the C3H gene family is an important force for functional divergence to stress response, this may be why the *P. brtulaefolia* are used as rootstocks with fine comprehensive stress tolerance. These genes provided clues to the evolution of duplicated genes and stress tolerance improvement of *P. betulaefolia*.

### Excellent candidates for salt-tolerance improvement

In this paper, we identified some important candidate genes that were highly or specifically expressed after salt stress treatment; for example, in roots, *PbeC3H30* was significantly induced after 24 h of salt stress, and *PbeC3H59* responded to salt stress at 24 and 48 h (Fig. 6). Genes *PbeC3H2*, *PbeC3H30*, *PbeC3H47*, *PbeC3H67*, *PbeC3H96*, *PbeC3H77*, *PbeC3H88*, *PbeC3H92* and *PbeC3H113* were induced under salt stress in leaves, especially for *PbeC3H77*, *PbeC3H88* and *PbeC3H113* (Fig. 7). A number of studies indicated that C3H genes were involved in various development stage and different stress response. For example, previous studies revealed that CarC3H26 and 51 had higher expression during early stages of chickpea seed development (Pradhan et al., 2017). In rice, *OsC3H33*, *OsC3H37* and *OsC3H50* were induced by salt stress (Muhamman, Waqas & Asia, 2010). Another C3H gene in rice, *OsC3H12* positively and quantitatively regulates rice resistance to bacterial leaf blight caused by *Xanthomonas oryzae pv oryzae*, which is likely associated with the jasmonic acid dependent pathway (Deng et al., 2012). These genes involved in salt stress response provide fine candidates for salt-tolerance improvement in *P. betulaefolia*.

**Figure 8:**
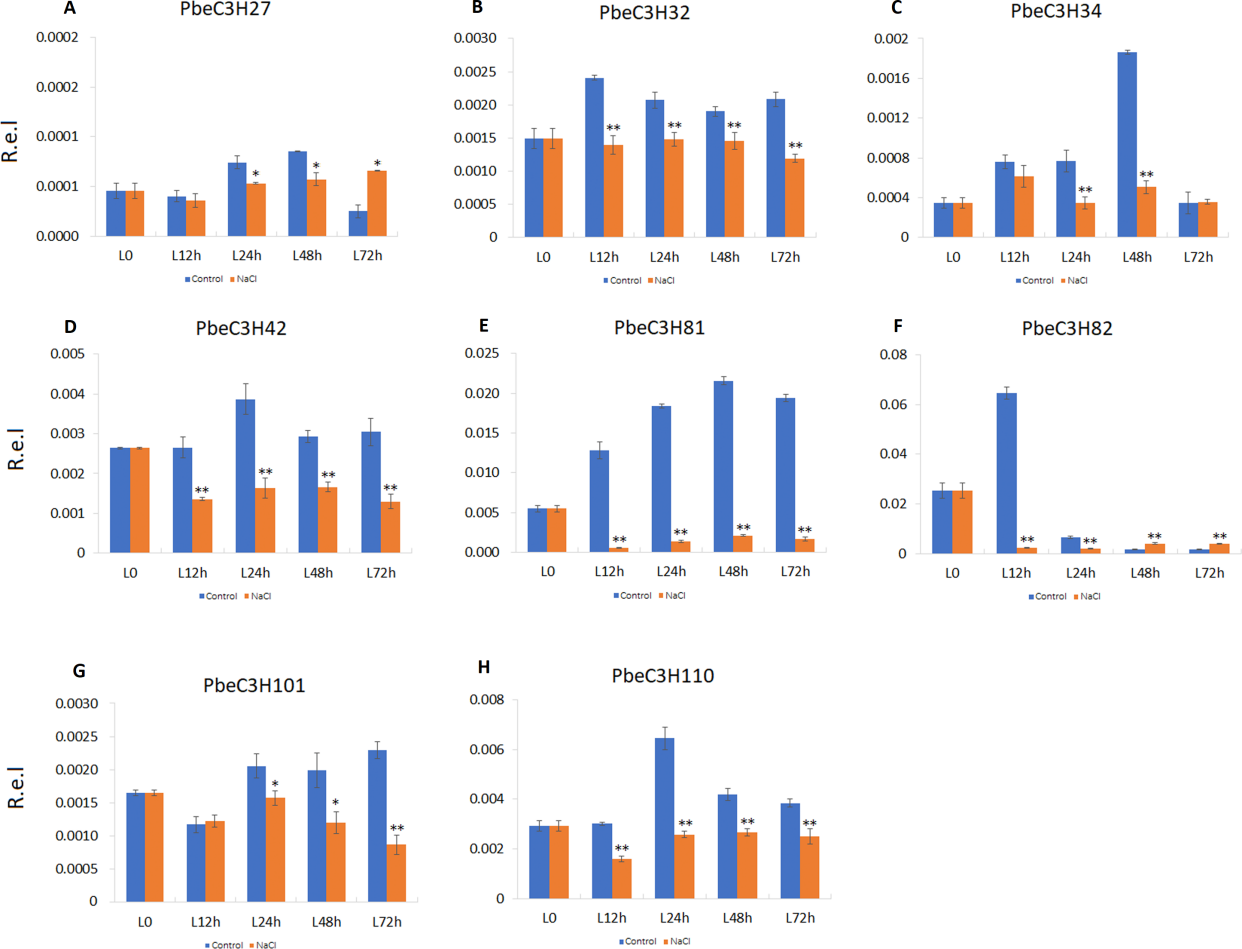
Expression profiles of eight *C3H* genes repressed by salt stress in leaves. (A–H) Expression for each *PbeC3H* gene. ((A) PbeC3H27; (B) PbeC3H32; (C) PbeC3H34; (D) PbeC3H42; (E) PbeC3H81; (F) PbeC3H82; (G) PbeC3H101; (H) PbeC3H110). Relative expression was calculated using the 2^−ΔΔ*CT*^ method. R.e.l indicates relative expression level. The asterisk and double asterisks represent significant differences at the levels of 0.05 and 0.01, respectively.

We also found that some *PbeC3H* genes were repressed under salt stress or sensitive to salt stress; for example, *PbeC3H27*, *PbeC3H34*, *PbeC3H42*, *PbeC3H82*, *PbeC3H81*, *PbeC3H32*, *PbeC3H101* and *PbeC3H110* (Fig. 8). These genes were significantly repressed after salt stress treatment at one or more tested time point. Notably, expression of *PbeC3H42 PbeC3H81*, *PbeC3H32* and *PbeC3H110* were significantly reduced after salt stress treatment. Especially for *PbeC3H81*, this was about 10% of the expression of controls (Fig. 8). These genes response to salt stress or functionalized in mental ions transport and metabolism (Table S4) are also excellent candidates for studying the mechanism of salt response in *P. betulaefolia*.

## Conclusions

The C3H-type Znf family transcription factors play vital roles in plant development and response to biotic and abiotic stresses. We performed the first genome-wide analysis of the C3H family genes in *P. betulaefolia* and conducted a detailed investigation of their classification, structure, gene evolution and expression profiles under salt stress. All the 117 PbeC3H genes were classified into 12 groups based on the organization of various characteristic domains and mapped onto 17 chromosomes. The identification and classifications were supported by structural characteristics of the genes and proteins, as well as by phylogenetic analysis. There were 35 and 37 pairs of paralogous genes in the *P. betulaefolia* and apple genome, respectively, and 87 pairs of orthologous genes between them. Except for one orthologous pairs *PbeC3H66* & *MD05G1311700* which had undergone positive selections, the other C3H genes had undergone purifying selection. And the C3H genes expanded in *P. betulaefolia* than that in *P. bretschneideri*. Expression profiles showed that high salinity stress could influence the expression level of C3H genes in *P. betulaefolia* and we found genes response to stress contained relative complex gene structure. Genes induced or inhibited by salt could be used as excellent candidates for further stress response research. The present study provides a foundation for understanding the complex functions of the *PbeC3H* gene family and will facilitate studies of them to salt stress response in *P. betulaefolia* salt tolerance improvement.

##  Supplemental Information

10.7717/peerj.9328/supp-1 Table S1 Raw expression data of the four genes in Fig. 6

10.7717/peerj.9328/supp-2 Table S2 Raw expression data of the four genes in Fig. 7

10.7717/peerj.9328/supp-3 Table S3 Raw expression data of the four genes in Fig. 8

10.7717/peerj.9328/supp-4 Table S4 GO analysis of the C3H genes

10.7717/peerj.9328/supp-5 Supplemental Information 5 qRT-PCR expression data

## Additional Information and Declarations

### Data Availability

The following information was supplied regarding data availability: Raw data is available as a Supplemental File.
